# Parental Representations and Emotional Availability: The Case of Children with Autism and Severe Behavior Problems

**DOI:** 10.1007/s10803-024-06629-3

**Published:** 2024-11-13

**Authors:** Efrat Sher-Censor, Moria Harel, David Oppenheim, Adi Aran

**Affiliations:** 1 School of Psychological Sciences and the Center for the Study of Child Development, Rabin Building, The University of Haifa, 199 Aba Khoushy Ave., Mount Carmel, 3498838 Haifa, Israel; 2 Neuropediatric Unit, Shaare Zedek Medical Center, 9103102 Jerusalem, Israel; 3 Faculty of Medicine, Hebrew University of Jerusalem, 9112102 Jerusalem, Israel

**Keywords:** Autism, Behavior problems, Emotional availability, Fathers, Five minute speech sample, Mothers, Resolution with the child’s diagnosis

## Abstract

Studies suggest that parents’ emotional availability (EA) is associated with children’s wellbeing, including in the case of children with autism. Our study extended prior research by examining the role of parents’ representations in fostering parental EA and by focusing on fathers and on children with autism and severe behavior problems. We expected that parents’ positive representations would be associated with higher EA and compared mothers’ and fathers’ representations and EA. Participants were 79 mothers and 69 fathers (child age range = 61–173 months, 21.95% girls). Representation assessments included the Reaction to Diagnosis Questionnaire, tapping resolution with respect to the child’s diagnosis, and the coherence and positive comments in parents’ Five Minute Speech Samples about the child. Parents’ EA was coded from parent–child play interactions. Controlling for children’s autism symptoms and adaptive functioning, mothers’ resolution with respect to the child’s diagnosis and positive comments (but not coherence) were associated with their EA, and fathers’ coherence (but not positive comments and resolution) was associated with their EA. Mothers expressed more positive comments than fathers, and the resolution and EA scores of mothers and fathers were significantly correlated. Our results highlight the importance of considering both parents’ representations and EA when studying and working with families of children with autism and severe behavior problems.

**Supplementary Information:**

The online version contains supplementary material available at 10.1007/s10803-024-06629-3.

## Introduction

Parents’ emotional availability (EA) refers to sensitive behaviors towards the child without intrusiveness or hostility (Biringen et al., 2014). It contributes to the wellbeing and development of typically developing children and children with autism (Biringen et al., 2022; Cossette-Côté et al., 2022). Studies of typically developing children further suggest the representations that parents construct about the child, namely parents’ thoughts, feelings, and expectations of their child, may shape their EA (e.g., Koren-Karie et al., 2002; Sokolowski et al., 2007). Only a few studies have examined this association in the context of parents of children with autism, however (Hutman et al., 2009; Oppenheim et al., 2012, 2024; Sher-Censor et al., 2017; Wachtel & Carter, 2008).

The current study joined this body of work and aimed to address three gaps. First, three aspects of parents’ representations are thought to be related to EA: *resolution* or coming to terms with the child’s autism diagnosis (e.g., Oppenheim et al., 2024); *coherence* when narrating about the child, i.e., the ability to provide a clear, consistent, and multidimensional portrayal of the child (e.g., Sher-Censor et al., 2017); and *positive comments* in parents’ narratives that reflect warmth and appreciation toward the child (Pasalich et al., 2011). However, none of the studies examined all three representation aspects within a single study. In addition, previous findings on each aspect and their associations with EA were inconsistent (e.g., Sher-Censor et al., 2017 documented this link, whereas Hutman et al., 2009 failed to find it).

Second, despite growing evidence of the unique contribution of father-child relationships to children’s development in general (Paquette et al., 2020) and those with autism in particular (Rankin et al., 2019), studies of parental representations and EA towards children with autism focused for the most part on mothers and did not include fathers. Finally, although more than 50% of the children with autism show significant behavior problems, studies of parental representations and EA did not consider these problems. Behavior problems include externalizing behaviors, such as engaging in harmful and dangerous activities and frequent and prolonged temper tantrums, and internalizing behaviors, such as chronic anxiety and depressed mood (Salazar et al., 2015; Soke et al., 2018). Such behaviors may negatively affect children by disrupting daily functioning and limiting opportunities for learning and social interactions (Maskey et al., 2013). They can also harm families by weakening family cohesion (i.e., shared affection and support) and increasing parental psychological distress (Yorke et al., 2018). Thus, studying parental representations and EA in the context of children with autism and severe behavior problems is both understudied and may have important clinical implications.

### Parental EA

Parental EA towards their child involves being sensitive to the child's signals, providing appropriate structure to the interaction, and respecting the child's autonomy. By being emotionally available, parents mediate the external world to the child, scaffold the child’s exploration, and help the child soothe when distressed (Biringen et al., 2014). Numerous studies have shown that parental EA promotes the development and well-being of typically developing children (Biringen et al., 2014, 2023).

Being emotionally available to children with autism may be particularly challenging for parents because children with autism have difficulties in communication and social interaction. Indeed, research suggests that children’s autism symptoms (Dolev et al., 2009) and adaptive functioning (Sher-Censor et al., 2017) affect maternal EA. Importantly, similarly to typically developing children, children with autism benefit from emotionally available parenting. Mothers’ EA fosters secure attachment of children with autism (Cossette-Côté et al., 2022) and contributes to children’s cognitive and language development (Baker et al., 2010; Siller & Sigman, 2002) above and beyond children’s autism symptoms and adaptive functioning. Thus, it is important to study factors (such as parental representations) that may shape parents’ EA towards children with autism.

In addition, there is a need to study paternal, and not only maternal EA. Studies of typically developing children suggest mothers and fathers may conduct somewhat different types of interactions with their children. Fathers are generally more likely than mothers to encourage their children to explore, take risks, and be active (Paquette et al., 2020), and may show somewhat lower EA than mothers (Biringen et al., 2022). There is relatively little research on fathers of children with autism (Rankin et al., 2019). We are aware of only three studies that examined the EA of these fathers. One reported no differences between the EA of mothers and fathers (Bentenuto et al., 2020). The second indicated no differences between mothers’ and fathers’ sensitivity, which is a core aspect of parental EA (Oppenheim et al., 2024). The third focused only on children with autism without intellectual disability and showed differences between mothers and fathers in two aspects of EA. Mothers were more sensitive and provided more appropriate structuring to their children than fathers. Furthermore, this study pointed to the importance of assessing both parents’ EA because the EA of mothers and fathers had distinct associations with children’s emotion regulation skills (Hirschler-Guttenberg et al., 2015). Interestingly, the studies did not report the concordance of EA levels between mothers and fathers. Such an association could be expected because the parents are interacting with the same child and because they may be impacted by observing the other parent’s interactions with the child. Therefore, the current study examined not only mean-level differences between parents’ EA but also the concordance between parents.

### Resolution with Regard to the Child’s Diagnosis

As noted above, we examined the links between three aspects of parents’ representations and their EA. The first aspect was resolution with regrad to the child’s diagnosis. The extent to which parents have come to terms with their child’s medical or developmental diagnosis and resolved their negative feelings regarding that diagnosis is thought to play a central role in their relationship with the child (Marvin & Pianta, 1996; Oppenheim et al., 2009). Parents often respond to receiving such diagnoses for their children with powerful negative emotions. Resolution with regard to the diagnosis indicates parents’ processing of these emotions, acknowledging the challenges involved in parenting the child, and revising expectations regarding the child in line with the diagnosis (Marvin & Pianta, 1996).

In the case of autism, up to 67% of parents might struggle with adapting to their children’s diagnosis even years after receiving it (Sher-Censor & Shahar-Lahav, 2022). Parents may be overwhelmed with grief or anger or, conversely, disengage and distance themselves from the child’s challenges. These reactions do not only impact parents internally, however. The hypothesis is that they may lower parental EA and be expressed in insensitive, intrusive, or hostile responses to the child (Marvin & Pianta, 1996; Sher-Censor & Shahar-Lahav, 2022).

Yet, support for these notions is inconsistent. Three studies indicated that mothers’ resolution with regard to their child’s autism diagnosis was associated with their higher EA (Sher-Censor et al., 2017) or sensitivity towards their child (Oppenheim et al., 2012, 2024), as expected. However, two other studies that assessed constructs similar to EA, synchrony and supportive engagement, did not find significant links (Hutman et al., 2009; Wachtel & Carter, 2008).

In addition, less is known about fathers’ resolution with respect to the child’s autism diagnosis. A few studies reported no difference between mothers’ and fathers’ resolutions (Milshtein et al., 2010; Oppenheim et al., 2024; Yirmiya et al., 2015). One of these studies also reported that in 53% of the families, parents showed a concordance in their resolution status (Milshtein et al., 2010). To our knowledge, only one study examined the links between fathers’ resolution with their children’s autism diagnosis and their sensitivity towards the child and did not find a significant link between them (Oppenheim et al., 2024). Thus, more research is needed.

### Coherence in Narratives About the Child

The second aspect of parents’ representations we assessed was coherence, which refers to the extent to which parents portray their child in a clear, consistent, multifaceted, and well-supported manner (Slade et al., 1999). Incoherence is expressed in contradictory descriptions, impoverished or one-sided portrayals, overwhelmed concern, rejection of the child, or separateness issues (Koren-Karie et al., 2002). Such difficulties in narrating coherently are thought to underlie low EA, as expressed in responses that are not attuned to the child’s signals or disengagement from the child (Sher-Censor & Yates, 2015). Several studies supported these notions in mothers of typically developing children (e.g., Korja et al., 2010; Slade et al., 1999; Sokolowsky et al., 2007). We are aware of only one study documenting such a link in mothers and children with autism (Sher-Censor et al., 2017). To our knowledge, the current study was the first to examine it among fathers of children with autism.

### Positive Comments in Narratives About the Child

The third aspect of parents’ representations of their children that is thought to foster their EA is positive comments. These involve positive remarks about the child’s characteristics and behavior, praising the child, and expressing affection, love, and appreciation (Pasalich et al., 2011). The positive perception of the child can promote engagement with the child, empathy when the child is showing challenging behaviors, and expression of warmth when interacting with the child, all aspects of parental EA (Pasalich et al., 2011). Expressing positive comments about the child might be particularly challenging for parents of children with autism and severe behavior problems.

Elevated levels of behavior problems in children with autism are associated with fewer positive comments in their mothers’ and fathers’ narratives (Baker et al., 2019; Hickey et al., 2020; Lorang et al., 2022). Relatedly, parents of children with autism and parents of children with high levels of externalizing behavior problems expressed fewer positive comments regarding the child than parents of children with other developmental diagnoses, such as cerebral palsy, Down syndrome, developmental delay, and mood disorders (De Clercq et al., 2022; Pasalich et al., 2011). Nevertheless, studies of mothers of children with clinical levels of externalizing behavior problems (without a diagnosis of autism) have documented that expression of more positive comments was associated with mothers’ scaffolding of less harsh and more supportive interactions with their children (Weston et al., 2017). We are not aware of studies on the links between positive comments and EA of mothers or fathers in the context of children with autism.

### The Current Study

The study focused on parents of children with autism and severe behavior problems. It assessed parents’ EA and three aspects of their representations: resolution with regard to the child’s diagnosis, coherence when narrating about the child, and positive comments in these narratives. Our first goal was to examine the links between parents’ representations and their EA. We hypothesized that for both mothers and fathers, higher resolution with respect to the child’s diagnosis, higher coherence, and expression of more positive comments in their narrative about their child would each be associated with higher EA. The hypothesis was tested while controlling for children’s autism characteristics and adaptive behavior.

Our second goal was to explore similarities and differences between mothers and fathers in their representations and EA. We did not form an a priori hypothesis as research on this issue is limited and provides inconsistent results (e.g., Bentenuto et al., 2020 vs. Hirschler-Guttenberg et al., 2015).

## Method

### Participants

Eighty-two Israeli families of children diagnosed with autism and severe behavior problems participated in the study. Inclusion criteria included children with established autism diagnosis based on the DSM-5 criteria (American Psychiatric Association, 2013) confirmed by the Autism Diagnostic Observation Schedule, Second Edition (ADOS-2; Lord et al., 2012) assessment; behavioral problems as indicated by a score of 18 or higher on the Aberrant Behavior Checklist-Irritability Subscale (ABC-I; Aman & Singh, 1986); and parents who spoke Hebrew fluently.

Data were collected from 79 mothers and 69 fathers and their children. In 66 families, both parents took part in the study; in 13 families, only the mother participated; and in 3 families, only the father participated. Only one child from each family participated in the study. See parents’ and children’s characteristics in Table 1. Both parents signed a consent form. For children capable of understanding the study procedures as perceived by their parents or the primary investigator, the parents and the primary investigator explained the study aims and methods using appropriate language and obtained their written assent.

**Table 1:** Parents and children’s characteristics

	Mean/n	Range	SD/%
Child gender			
Female	18		21.95%
Male	64		78.15%
Child age (in months)	116.90	61–173	30.13
Child birth order	1.39	1–9	1.75
Child ADOS CSS score			
8–10 (severe autism symptoms)	62		76.54%
4–7 (mild to moderate autism symtpoms)	19		23.46%
Child adaptative behavior score			
Below 70 (low adaptative behavior)	71		86.59%
70 or above	11		13.41%
Parents’ self identified religion			
Jewish	80		97.56%
Non-Jewish	2		2.44%
Parents marital status			
Married	76		92.68%
Divorced	3		3.66%
Single parent	3		3.66%
Mothers’ years of education	15.13	10–24	2.81
Fathers’ years of education	15.10	7–22	2.78
Number of children with autism in the family			
One child	55		67.07%
More than one child	27		32.93%

### Measures

#### Children’s Characteristics

##### Autism Symptoms

*The Autism Diagnostic Observation Schedule, Second Edition* (*ADOS-2*; Lord et al., 2012) was used to evaluate the severity of children’s autism symptoms. The ADOS-2 is considered the gold-standard assessment of autism severity (Gotham et al., 2009; Wiggins et al., 2017). It assesses social interaction, communication, play, and imaginative use of materials through an interaction between the child and a trained professional. It consists of four modules, one of which was administered to the child based on the child’s level of expressive language. Module 1 was administered to 60.98% of the children. Module 2 was administered to 14.63% of the children, and module 3 was administered to 23.17%. The diagnostic algorithm of the ADOS was used (Gotham et al., 2007), and the Calibrated Severity Score (CSS; Gotham et al., 2012) was calculated. The CSS provides a standardized measure of autism symptom severity, ranging from 1 to 10. Scores of 8 to 10 reflect severe symptoms, scores 4 to 7 reflect mild to moderate symptoms, and scores 1 to 3 indicate a low level of symptoms (Lord et al., 2012). ADOS-2 sensitivity and specificity range across different modules and populations (see Dorlak et al., 2018 review reporting pooled sensitivity estimates of 0.77–0.90 and pooled specificity estimates of 0.62-0.090 across modules and studies). Nevertheless, its CSS exhibits strong psychometric properties, including high internal consistency and test–retest reliability (Janvier et al., 2022; Wiggins et al., 2017). ADOS-2 was coded by two independent trained researchers. Inter-rater reliability was calculated for 22% of the sample, and the average agreement was 85.6%.

##### Adaptive Behavior

The Vineland Adaptive Behavior Scales (VABS; Sparrow et al., 2005) assessed children's adaptive behavior. The VABS is a structured parental interview for assessing children’s adaptive skills in the domains of socialization (i.e., functioning in social situations), communication (i.e., how well children listen, understand, and express themselves through speech), and daily living skills (i.e., performance of practical, everyday tasks of living that are appropriate in the school setting). For children up to age seven, the assessment also included their fine and gross motor skills. The VABS yields standardized scores with a mean of 100 and *SD* of 15. A score below 70 reflects low adaptive behavior. The VABS has been extensively used in studies of children with autism, including in Israel (e.g., Dolev et al., 2009).

#### Parental Measures

##### Parental EA

Parents’ EA towards their children was assessed through videotaped parent–child interactions, which included seven minutes of free play, in which the parent and the child were invited to play together with various toys, and seven minutes of social play, in which dyads were invited to play together without any toys. Interactions were coded using the four parental 7-point scales of the *Emotional Availability Scales—4th Edition (EAS; *Biringen, 2008*).* The *Sensitivity* scale assesses the parent’s attunement and responsiveness to the child's signals while expressing warmth and emotional connectedness. It ranges from 1 (“Highly insensitive”) to 7 (“Highly sensitive”). The *Structuring* scale assesses the appropriate scaffolding of the child’s play by following the child’s lead and setting limits to the child’s behavior if needed. It ranges from 1 (“Non-optimal structuring”) to 7 (“Optimal structuring”). The *Nonintrusiveness* scale assesses the absence of parents’ over-direction, overstimulation, interference with the child's activities, and/or overprotection. It ranges from 1 (“Intrusive”) to 7 (“Nonintrusive but emotionally present/available”). The *Nonhostility* scale assesses the absence of parents’ dissatisfaction, impatience, or belittling of the child. It ranges between 1 (“Markedly and overtly hostile”) to 7 (“Non-hostile”).

The EAS are widely used, including in studies of children with autism (for review, see Biringen et al., 2014, 2022). Six trained coders conducted the EAS coding. They did not participate in data collection and remained blind to all other data, including the parent and child scores from other measures and the EAS coding results of other episode assessments for the same family. A coder that coded one of the interactions between a parent and child (e.g., mother–child free play) did not code their second interaction (e.g., mother–child social play) nor the other parent’s interactions with the child (e.g., father-child free play and father-child social play). Inter-rater reliability of mothers’ and fathers’ EAS based on 25% of the observations was excellent and ranged between ICC = 0.74 to ICC = 0.95, median = 0.85. The four EAS scores of the free and the social play episodes were strongly correlated both within each episode and across episodes for both mothers and fathers (*r*_*range*_ = 0.65—0.94, all *p*’s < 0.001), and therefore, the scores were averaged to form a parental EA score. Higher scores reflected higher EA.

##### Parents’ Resolution with Respect to the Child’s Diagnosis

Parents completed the *Reaction to Diagnosis questionnaire (RDQ*; Dan Ram-On & Sher-Censor, 2016) to assess their resolution with respect to their child’s diagnosis. The RDQ consists of 42 items reflecting resolution (e.g.., “I believe that my family and me can cope with my child’s difficulties and help him/her”) or lack of resolution (e.g., “I am convinced there is a mistake with respect to my child's diagnosis”). Parents were asked to rate each item on a scale ranging from 1 ("strongly disagree") to 5 ("strongly agree"). After reverse coding of items reflecting a lack of resolution, a mean score was calculated. Higher scores indicated better resolution. In a study of 75 mothers of children diagnosed with cerebral palsy or developmental delay (Sher-Censor et al., 2020), the RDQ demonstrated good internal consistency and was validated against the gold-standard interview assessment of parental resolution, the Reaction to Diagnosis Interview (RDI; Pianta & Marvin, 1992). Specifically, mothers classified as 'resolved' on the RDI had significantly higher RDQ scores than those classified as 'unresolved.' Additionally, the area under the ROC curve analysis indicated that the RDQ effectively discriminated between resolved and unresolved mothers, as classified by the RDI. Moreover, the patterns of associations between both the RDI and RDQ with family characteristics—such as the child’s functioning level, time since diagnosis, and maternal depressive symptoms—were similar. The RDQ's internal reliability in the current study was good (Cronbach’s Alpha _mothers_ = 0.86; Cronbach’s Alpha _fathers_ = 0.85).

##### Coherence and Positive Comments in Parents’ Narratives

To evaluate parents’ coherence and positive comments when narrating about the child, parents were audio-recorded while speaking for five uninterrupted minutes about “what kind of person their child is”, and “how the two of them get along” using the FMSS procedure (FMSS; Magaña et al., 1986). The FMSS was transcribed verbatim. Identifying information was removed from the transcripts, including parental gender. Transcripts were subsequently coded by coders who did not participate in data collection and were blind to parent and child scores from other measures.

*FMSS-Coherence*. To assess the coherence of parents’ narratives, transcripts were coded using the FMSS-Coherence scales (Sher-Censor & Yates, 2012), which were adapted from the Insightfulness Assessment (Koren-Karie & Oppenheim, 2004; Koren-Karie et al., 2002). Transcripts were rated on six 7-point scales tapping salient dimensions of coherence, including *Focus, Elaboration, Separateness, Concern/worry, Acceptance/rejection,* and *Complexity* (see Sher-Censor & Yates, 2015, for further detail). Based on the scale scores and an evaluation of the overall internal consistency of the FMSS, a global coherence rating was assigned on a 7-point scale. The scale ranged from 1 (The narrative does not provide a description of the child); through 5 (The parent provides a multidimensional portrayal of the child, statements are well-supported, but a small portion of the narrative lacks coherence); to 7 (The narrative is consistent, multifaceted; it conveys an individualized and balanced portrayal of the child).

The FMSS-Coherence scale has demonstrated excellent inter-rater reliability in past research (e.g., ICC = 0.82–0.95; Foley et al., 2019; Vashi et al., 2024; Kappa = 0.83–1.00, Sher-Censor et al., 2016, 2017). It has also shown theoretically consistent concurrent validity (e.g., Foley et al., 2019) and predictive validity (e.g., Sher-Censor et al., 2016; Vashi et al., 2024). For example, it has been linked with mothers’ EA toward their children with autism (Sher-Censor et al., 2017) and predicted reduced externalizing behavior problems, increased ego-resilience, and improved peer acceptance in children with self-regulation difficulties during the transition from preschool to school (Sher-Censor et al., 2016). Recent findings indicate that improvements in parents’ coherence following a 10-week CBT intervention for their child predicted reductions in behavioral symptoms of children with autism (Vashi et al., 2024), further supporting the scale's predictive validity.

*FMSS-Positive Comments*. Coding of parents’ positive comments was carried out using the FMSS-Expressed Emotion coding outlined by Magaña-Amato (1993) and adapted to parents by Wamboldt et al. (2000). The Expressed Emotion coding system includes several additional indices (e.g., emotional overinvolvement and criticism). However, these indices were not used in the current study due to their similarity with the FMSS-Coherence scales. An index of positive comments was formed by counting the number of positive remarks regarding the child (e.g. “she is very smart”) and remarks reflecting love and appreciation (e.g., “I love him very much”). Support for the validity of this coding was provided by its links with observed interaction with children with typical development (Weston et al., 2017) and their bidirectional links with children’s functioning and behavior problems among children with autism (Hickey et al., 2020). Inter-rater reliability of the coherence and positive comments coding was established in 30% of the cases and was excellent (ICC _coherence_ = 0.84; ICC _positive comments_ = 0.95). Disagreements between coders were resolved through discussion.

### Procedure

The study was part of a larger project examining the effects of Cannabinoid treatment on children diagnosed with autism and severe behavior problems (Aran et al., 2021). Families were recruited from across the country by neurologists and child psychiatrists. Data collection for this study took place in the baseline phase and was conducted in the Hospital clinic. The visit lasted about three hours. A trained developmental psychologist administered the ADOS-2 (Lord et al., 2012) to assess children’s autism symptoms. A child neurologist interviewed the parents, confirmed the diagnosis of ASD based on DSM-5 criteria, and assessed the severity of children’s behavioral problems using the Clinical Global Impression – Severity scale (Guy, 1976) with anchoring instructions. This assessment confirmed that all children showed at least moderate levels of externalizing and internalizing behavior problems. One of the parents (88.37% mothers) was interviewed using the VABS (Sparrow et al., 2005) to evaluate children’s adaptive behavior.

To measure parents’ coherence and positive comments, parents were invited to narrate about the child using the FMSS (Magaña et al., 1986). Parents then completed the RDQ (Sher-Censor et al., 2020) to assess their resolution with respect to the child’s diagnosis. Interviews with each parent were held in a quiet room while the other parent was videotaped in another room playing with the child with toys (i.e., “free play”) and without toys (i.e., “social play”). Types of play (free/social) and parents' order (father/mother) were counterbalanced. Interviews were audiotaped, and observations were videotaped. The study was approved by the Institutional Review Board of the Shaare Zedek Medical Center and the Israeli Ministry of Health.

### Data Preparation and Analytic Plan

Data analyses were conducted using SPSS 27. Unless otherwise specified, the significance level was set at < 0.05. All continuous variables were sufficiently normal to render parametric statistics valid (Afifi et al., 2007). Of the 79 mothers, three did not participate in observed interaction with the child, three did not complete the RDQ, and two did not provide an FMSS. In addition, of the 69 fathers, three did not participate in observed interaction with the child, three did not complete the RDQ, and one did not provide an FMSS. Except for one father who did not want to be videotaped interacting with their child and another who did not wish to complete the RDQ, other missing data were due to technical errors. One child did not cooperate with the developmental psychologist and thus did not complete the ADOS-2 assessment.

Preliminary analyses included correlations and independent-sample *t*-tests to examine the associations between family demographics (e.g., child gender and parents’ years of education) and children’s autism symptoms and adaptive behavior and the study variables. These analyses informed the inclusion of covariates in the subsequent analyses. The intercorrelations among the study variables were examined to test the hypothesis that parents’ representations would be associated with their EA, followed by four linear regressions, two predicting mothers’ EA and two predicting fathers’ EA. In all regression models, relevant covariates were entered in the first block, followed by one of the representational measures. Two regression models included in the second block mothers’ or fathers’ resolution scores. Two regression models included in the second block mothers’ or fathers’ FMSS scores. Finally, paired-sample *t*-tests and Pearson correlations were conducted to examine differences and concordance between mothers and fathers in the study variables.

## Results

### Preliminary Analyses

As can be seen in Table 1, most children showed severe autism symptoms and low adaptive behavior, with no gender differences observed, χ^2^ (1) = 3.07, p = 0.080, and χ^2^ (1) = 0.11, *p* = 0.745. Table 2 shows descriptive statistics of study variables. Independent sample *t*-tests indicated two significant effects of child gender. Mothers of girls were more resolved with regard to their daughters’ diagnosis than mothers of boys, and fathers of girls were more coherent than fathers of sons. For further details about differences in study variables by child gender, see Supplementary Table 1. Parents’ years of education, child age, and whether there were other children with autism in the family were not significantly correlated with study variables (*p*’s > 0.095), except for fathers’ years of education, which was associated with their resolution with respect to the diagnosis (*r* = 0.24, *p* = 0.049). Hence, child gender and fathers’ years of education were included in subsequent analyses.

**Table 2:** Descriptive statistics (N = 66—77)

Study variables	N	Mean	Range	SD
Mothers’ resolution with resepct to the child’s diagnosis	76	3.77	2.85–4.67	.42
Mothers’ coherence	77	4.13	2–7	1.21
Mothers’ positive comments	77	4.27	0–12	3.20
Mothers’ emotional availability	76	5.13	2.50–6.75	.96
Fathers’ resolution with respect to the child’s diagnosis	66	3.83	3.05–4.69	.36
Fathers’ coherence	68	4.15	1–7	1.43
Fathers’ positive comments	68	2.65	0–9	2.49
Fathers’ emotional availability	66	4.92	1.94–6.47	1.09

Differences in study variables by the severity of children’s autism symptoms and adaptive behaviors are presented in Supplementary Table 2 and Supplementary Table 3. As shown in these Tables, the severity of children’s autism symptoms was not associated with study variables except for parents’ EA. Mothers and fathers showed lower EA towards children with severe autism symptoms than towards children with moderate autism symptoms. Children’s adaptive behavior was not associated with study variables except for the following. Fathers of children with low adaptive behavior reported lower resolution with respect to the child’s diagnosis than fathers of children with high adaptive behavior. In addition, mothers and fathers showed lower EA toward children with low adaptive behavior than toward children with high adaptive behavior. Thus, children’s severe versus moderate autism symptoms and children’s low versus high adaptive behavior were included in subsequent analyses.

### Associations Between Parents’ Representations and Their EA

Table 3 presents correlations between study variables. Mothers’ resolution with regard to the child’s diagnosis and their positive comments, but not coherence, were each significantly correlated with their EA. Fathers’ resolution with regard to the child’s diagnosis and coherence, but not their positive comments, were each significantly correlated with their EA.

**Table 3:** Inter-Correlations of Study Variables (N = 66—77)

	1	2	3	4
1. Parent’s resolution with respect to the child’s diagnosis	.27*	.08	.26*	.26*
2. Parent’s coherence	.23	**.15**	.32**	.09
3. Parent’s positive comments	.17	.51**	**.07**	.33**
4. Parent’s emotional availability	.28*	.29*	.15	.51***

Correlations for mothers are presented above the diagonal, correlations for fathers are below the diagonal, and correlations between mothers and fathers are presented in bold on the diagonal

*p < .05. **p < .01. ***p < .001

Four regression analyses were conducted next, two predicting mothers’ EA and two predicting fathers’ EA. Preliminary analyses informed the inclusion of covariates in the first block of the regression models. The second block included parents’ resolution scores in two regression analyses (one predicting mothers’ EA and one predicting fathers’ EA), and parents’ FMSS scores in the other two regression analyses (again, one predicting mothers’ EA and one predicting fathers’ EA). Because correlation analyses indicated that mothers’ coherence was not associated with their EA and that fathers’ positive comments were not associated with their EA, regression analyses with these variables were not conducted.

As shown in Tables 4 and 5, controlling for children’s gender, children’s autism symptoms and adaptive behavior, mothers’ higher resolution with respect to the child’s diagnosis and more positive comments were associated with their higher EA. Controlling for children’s gender, autism symptoms, adaptive behavior and fathers’ years of education, fathers’ coherence was associated with higher EA. However, fathers’ resolution was not associated with their EA.

**Table 4:** Regression results of parents’ emotional availability on their resolution with respect to the child’s diagnosis

	Mothers’ emotional availability (*N* = 7*2*)			Fathers’ emotional availability (*N* = 6*3*)		
Variable in regression	β (entry)	β (final)	ΔR^2^	β (entry)	β (final)	ΔR^2^
Block 1			.20**			.25**
Children’s gender^a^	− .16	− .22		.21	.21	
Fathers’ years of education	–-	–		.20	.16	
Children’s autism symtpoms^b^	− .21	− .20		− .17	− .15	
Children’s adaptive behavior^c^	.29*	.26*		.30*	.28*	
Block 2			.05*			.02
Parents’ resolution		.24*			.14	
Total R^2^	.25			.27		
Final model	*F* (4, 67) = 5.54***			*F* (5, 57) = 4.13**		

*Note*. ‘Entry’ refers to beta coefficients for each predictor when first included in the model. ‘Final’ refers to beta coefficients after all predictors were entered, reflecting each predictor's effect while accounting for all other variables. ^a^Male = 0, Female = 1. ^b^ Mild to moderate autism symptoms = 0, Severe autism symptoms = 1. ^c^Low adaptive behavior = 0, High adaptive behavior = 1

* *p* < .05. **p < .01. *** p < .001

**Table 5:** Regression results of parents’ emotional availability on their FMSS scores

	Mothers’ emotional availability (*N* = 7*4*)			Fathers’ emotional availability (*N* = 65)		
Variable in regression	β (entry)	β (final)	ΔR^2^	β (entry)	β (final)	ΔR^2^
Block 1			.19**			.22**
Children’s gender^a^	− .16	− .16		.19	.10	
Fathers’ years of education	–	–		.20	.25*	
Children’s autism symptoms^b^	− .21	− .16		− .12	− .09	
Children’s adaptive behavior^c^	.28*	.27*		.32*	.35^**^	
Block 2			.07*			.08*
Mothers’ positive comments		.27*			–	
Fathers’ coherence		–-			.31*	
Total R^2^	.26			.30		
Final model	*F* (4, 69) = 5.91***			*F* (5, 59) = 5.17***		

*Note*. ‘Entry’ refers to beta coefficients for each predictor when first included in the model. ‘Final’ refers to beta coefficients after all predictors were entered, reflecting each predictor's effect while accounting for all other variables. ^a^Male = 0, Female = 1. ^b^ Mild to moderate autism symptoms = 0, Severe autism symptoms = 1. ^c^Low adaptive behavior = 0, High adaptive behavior = 1

*p < .05. **p < .01. ***p < .001

### Gender Differences and Concordance in Parents’ Representations and EA

Paired-sample *t*-tests indicated that there were no significant differences between mothers and fathers in their resolution with respect to the child’s diagnosis, coherence, and EA, *t* (62) = − 0.98, *p* = 0.329, Cohen’s *d* = 0.50; *t* (63) = − 0.25, *p* = 0.800, Cohen’s *d* = 1.72; and *t* (60) = 1.05, *p* = 0.300, Cohen’s *d* = 1.03 respectively. However, mothers expressed more positive comments (*Mean* = 4.13, *SD* = 3.22) than fathers (*Mean* = 2.70, *SD* = 2.52), *t* (63) = 2.89, *p* = 0.005, Cohen’s *d* = 3.94.

In addition, as shown in Table 3, higher maternal resolution was associated with higher paternal resolution, and higher maternal EA was associated with higher paternal EA. Yet, mothers’ and fathers’ coherence and positive comments were not significantly correlated (see Table 3).

## Discussion

The study was the first to evaluate three central aspects of parental representations: resolution with respect to the child’s diagnosis, coherence, and positive comments, and their associations with parental EA among mothers and fathers of children with autism and severe behavioral problems. The majority of the children in this sample showed severe core symptoms of autism and most of them exhibited low adaptive behavior. Nevertheless, parents varied in their representations of the children and in their EA when interacting with them. This suggests that parents' representations and EA are not solely determined by the severity of their children's condition. Parents can express resolution, coherence, and positive comments and remain emotionally available even when their children bring significant challenges to the relationship.

The first goal of this study was to examine the associations between parents’ representations and their EA. We found that the pattern of associations differed for mothers and fathers. Higher maternal resolution with regard to children’s diagnosis was, as hypothesized, associated with higher maternal EA. This result is consistent with prior studies among mothers of children with autism (Oppenheim et al., 2012, 2024; Sher-Censor et al., 2017) and extends them to the context of parenting children with autism who show severe behavior problems. Together, they support the theoretical notion suggested by Marvin and Pianta (1996) that experiencing positive changes in thoughts and feelings regarding the child’s diagnosis and revising the expectations from the child in line with the diagnosis facilitate attuned caregiving to the child.

Fathers’ resolution was significantly correlated with their EA; however, this association became non-significant after controlling for paternal education and children’s adaptive behavior. We are aware of two previous studies that examined this link. One involved fathers of children with autism (Oppenheim et al., 2024), and the other focused on fathers of children with mild intellectual disability (Barak-Levi & Atzaba-Poria, 2015). Neither study found a significant bivariate association. Further research is needed to determine whether resolution with respect to the child’s diagnosis has a greater influence on mothers' EA than on fathers' EA. Notably, in our study, the bivariate correlation between fathers’ resolution and EA was similar in magnitude to that observed for mothers. However, while children’s adaptive behavior was associated with both fathers’ resolution and EA, it was linked only to mothers’ EA and not to their resolution. This suggests that the father-child relationship may be more influenced by the child’s daily functioning than the mother–child relationship, a possibility that warrants further exploration.

The associations between parents’ coherence and expressions of positive comments when narrating about the child and parental EA also varied by parental gender. The number of positive comments but not the coherence of mothers’ narratives was associated with their EA, whereas the coherence of fathers’ narratives but not positive comments were related to their EA. The link between parents’ coherent narratives and EA is one of the core notions of attachment theory (Main et al., 1985). According to this view, the ability to perceive the child in a multifaceted yet logically congruent way as expressed in the narrative, enables parents to respond flexibly and appropriately to their child’s signals. Several studies documented this association among mothers of typically developing children (e.g., Korja et al., 2010; Sokolowsky et al., 2007) and among of mothers of children with autism (Sher-Censor et al., 2017). Our study is the first to replicate it in the context of fathers of children with autism.

Failing to find an association between mothers’ coherence and EA in this study was unexpected. Interestingly, a recent study also found that fathers’ coherence but not mothers’ coherence was associated with sensitivity towards their young infants (Branger et al., 2022). In a similar vein, a study that focused on insightfulness to the internal world of the child in parents’ narratives found that this link was evident only among fathers but not among mothers of children with autism (Oppenheim et al., 2024). More research on mothers’ coherence and their EA towards children with autism is needed to clarify these inconsistent results.

Research has shown that mothers’ and fathers’ positive comments that reflect love and appreciation of the child were related to better parent–child interactions in the context of children with externalizing behavior disorder (without autism, Pasalich et al., 2011). The current study joins this body of work and suggests that more positive comments when narrating about the child are related to EA also among mothers of children with autism. The lack of such a link among fathers could reflect a floor effect, as fathers tended less than mothers to express positive comments, a result which will be further discussed below.

The second goal of this study was to explore similarities and differences in parents’ representation and in their EA. Mothers and fathers showed similar levels of EA and their EA scores were associated. The lack of gender differences in EA is in line with a previous study of the EA of parents of children with autism (Bentenuto et al., 2020) and a study of parents’ sensitivity towards their children with autism (Oppenheim et al., 2024). This suggests that even though fathers may be less involved in the everyday caregiving of young children with autism compared to mothers (Rankin et al., 2019), it does not necessarily lead to a diminished capacity to engage in an emotionally available way with their children. It is also possible that the lack of differences between mothers and fathers and the concordance in parental EA are because parents observe each other’s behavior and, over time, adopt similar responses to their child.

We also did not find significant differences between mothers and fathers in their resolution with respect to their child’s diagnosis, which is consistent with past research (Milshtein et al., 2010; Oppenheim et al., 2024; Yirmiya et al., 2015). It seems that fathers are not different than mothers in their ability to reflect and report their thoughts and feelings regarding the child’s autism diagnosis and in individual differences in the extent to which they experience positive changes in these internal states and succeed in revising their expectations of the child in light of the diagnosis. As for the association between mothers’ and fathers’ resolution, only one of the above three studies went beyond exploring gender differences and examined the concordance between parents (Milshtein et al., 2010). The study revealed only a 53% concordance using the dichotomized classification of parental resolution as resolved versus unresolved (Marvin & Pianta, 1996), although they did not examine whether this effect was significant. In our study we found a significant yet weak correlation between the continuous resolution scores of mothers and fathers. This is similar to the findings of Milshtein et al. (2010). It suggests that although mothers and fathers in some families were similar in the extent to which they have come to terms with their child’s diagnosis, this association was far from full. The continuous resolution measure we used (Sher-Censor et al., 2020) is likely to reveal more nuanced differences in parents’ resolution than the dichotomized classification commonly used and employed in Milshtein et al. (2010). It may allow future studies to shed light on the extent to which resolution with respect to the child’s diagnosis is an individual rather than a couple process.

A different pattern of results emerged with respect to the similarities and differences between mothers and fathers in their coherence and positive comments. No difference or concordance were found in the coherence of mothers and fathers when narrating about the child. To the best of our knowledge, this was the first study to examine the coherence among fathers of children with autism. Previous studies on mothers and fathers transitioning to parenthood reported a similar pattern. The studies did not find differences between mothers and fathers nor concordance between them when narrating regarding the infant (Branger et al., 2022; Foley et al., 2019; although in Branger's study the coherence of the narratives of mothers and fathers before birth were significantly correlated). Thus, although both parents were asked to describe the same child, their coherence appears to reflect their individual characteristics which are only partially related to the child. Future research may explore these characteristics, which may involve parents’ state of mind regarding their own attachment experiences (Cowan et al., 2009; Main et al., 1985).

As for positive comments, there was a significant difference between mothers and fathers: Mothers expressed more such comments than fathers, and there was no evidence of concordance between them. We are aware of one study that examined differences between mothers and fathers in their positive comments regarding their children with autism (Hickey et al., 2020). This study did not find differences and did not report examining the concordance between parents. If the gender differences in positive comments found in the current study are replicated, they may reflect a greater involvement of mothers in daily caregiving of their children (Rankin et al., 2019). This increased involvement may provide mothers more opportunities to experience their children’s strengths and competence, not only challenging behaviors, and foster mothers’ construction of more positive perceptions of their child. These speculations should be explored in future research. Supporting this, a recent study using a new semi-structured interview and content analyses found that mothers of children with autism more frequently mentioned themes of love, tenderness, involvement, and care, while fathers were more likely to emphasize limited time spent with the child (Moshe et al., 2024).

By examining both the coherence and positive comments in parents’ narratives, our study contributes to the broader literature on parents’ narratives regarding their children. The results indicated an association between coherence and positive comments for both mothers and fathers. This suggests that as part of building a congruent and multidimensional portrayal of the child, parents tend to express more positive remarks reflecting love and appreciation of the child. Importantly, the study also showed that coherence and positive comments are distinct qualities, each showing a unique pattern of links with parental gender, parental resolution with respect to the child’s diagnosis, and parental EA. Studies that stem from the attachment tradition highlight the importance of the coherence of adult narration around attachment themes and parenting as the hallmark of a secure child-parent relationship, with the specific content of the narratives awarded less importance (Main et al., 1985; Slade et al., 1999). Studies based on models of family climate and expressed emotion focus on the content of parental narratives, such as the number of positive comments, and do not measure narrative coherence (Sher-Censor & Yates, 2015). To the best of our knowledge, this was the first study that examined both aspects in the context of parenting children with a developmental diagnosis. Our findings highlight the value of considering both narrative features in future studies.

### Strengths and Limitations

The strengths of this study included the inclusion of fathers, collecting data from different informants (i.e., parents and researchers), and using different methods (observations, interviews, standardized tests, and questionnaires). Three limitations of the research are important for guiding future studies. First, the study measures were concurrent, and this precluded determining the direction of effect between parental representations and EA. The theoretical models of resolution with respect to the child’s diagnosis (Marvin & Pinata, 1996), narrative coherence (Main et al., 1985), and positive comments (Pasalich et al., 2011) postulate that parents’ representations shape their behavior. However, the opposite direction of effect is also possible, in which parents’ behavior when interacting with the child is likely to affect parents’ thoughts and feelings about the child and the child’s diagnosis (Hickey et al., 2020; Pasalich et al., 2011). Second, the study’s sample included children with severe behavioral problems. Although at least 50% of children with autism show severe behavior problems (Salazar et al., 2015; Soke et al., 2018), rendering the study findings very relevant, the findings cannot be generalized to families of children with autism who do not show severe behavior problems. Relatedly, most parents were Jewish (i.e., part of the majority), married, and had post-high school education. Generalization to parents who vary on these charactersitics awaits additional research. Third, a few studies of parents and typically developing toddlers found that parents were more emotionally available to their daughters than their sons (Bornstein et al., 2010; Harel et al., 2002; Lovas, 2005). This finding is thought to reflect parents’ socialization of their daughters to emphasize interpersonal relationships (Chodorow, 1989). Future studies with larger subsamples of girls with autism may explore whether such differences are evident in the context of EA towards children with autism and whether they are also apparent in parents’ representations.

### Conclusions

Despite these caveats, the findings highlight the added value of assessing the representations mothers and fathers construct about children and point to the importance of evaluating parenting at both the representational and behavioral levels (Main et al., 1985). This may be generally true in studies of the relationships between parents and children with autism but appears also to be the case with regard to children with autism and severe behavior problems. Even though severe behavior problems are likely to powerfully shape children’s relationships with their parents, the findings point to substantial variability in parent–child relationships, including in both parents’ representations and their behavior. The study may guide interventions with families of children with autism. It suggests that coming to terms with the child’s diagnosis and seeing the unique and positive characteristics of the child beyond the child’s autism characteristics, difficulties in adaptive behavior, and behavior problems may be valuable targets for screening and intervention efforts. Furthermore, the results regarding fathers suggest that they may be an under-utilized asset in early interventions with young children with autism, including those with severe behavior problems.

## Supplementary Information

Below is the link to the electronic supplementary material.

Supplementary file1 (DOCX 23 kb)

## Data Availability

Data supporting the findings are available from the corresponding author upon reasonable request.
