# Supplementary material for: Parental Representations and Emotional Availability: The Case of Children with Autism and Severe Behavior Problems
Source: J Autism Dev Disord. 2024 Nov 13;56(4):1489–502. doi: 10.1007/s10803-024-06629-3 (PMC12987863; doi:10.1007/s10803-024-06629-3)
Supplement: Supplementary file 1 — Supplementary file1 (DOCX 23 kb) [file 10803_2024_6629_MOESM1_ESM.docx]

**Supplementary material**

**Parental Representations and Emotional Availability: The Case of Children with Autism and Severe Behavior Problems**

**Supplementary Table 1**

*Parents’ Representations and Emotional Availability by Child Gender*

|  | Girls | | | Boys | | |  |
| --- | --- | --- | --- | --- | --- | --- | --- |
| Variable | n | *M* | *SD* | n | *M* | *SD* | t-test |
| Mothers’ emotional availability | 16 | 4.93 | 1.07 | 60 | 5.18 | .93 | .93 |
| Mothers’ resolution with child diagnosis | 17 | 3.98 | .38 | 59 | 3.72 | .42 | 2.30* |
| Mothers’ coherence | 16 | 3.94 | 1.05 | 61 | 4.18 | 1.25 | .73 |
| Mothers’ positive comments | 16 | 4.56 | 3.41 | 61 | 4.20 | 3.17 | -.40 |
| Fathers’ emotional availability | 14 | 5.35 | .82 | 52 | 4.81 | 1.13 | -1.70 |
| Fathers’ resolution with child diagnosis | 14 | 3.84 | .35 | 52 | 3.83 | .37 | 0.03 |
| Fathers’ coherence | 13 | 5.12 | 1.54 | 55 | 3.93 | 1.31 | -2.84** |
| Fathers’ positive comments | 13 | 3.38 | 2.75 | 55 | 2.47 | 2.42 | -1.19 |

**p* < .05. ***p* < .01.

**Supplementary Table 2**

*Parents’ Representations and Emotional Availability by the Severity of Children’s Autism Symptoms*

|  | Severe autism symptoms (ADOS ≥ 8) | | | Moderate autism symptoms  (ADOS < 8) | | |  |
| --- | --- | --- | --- | --- | --- | --- | --- |
| Variable | n | *M* | *SD* | n | *M* | *SD* | t-test |
| Mothers’ emotional availability | 57 | 4.97 | .99 | 18 | 5.63 | .72 | -2.58* |
| Mothers’ resolution with child diagnosis | 58 | 3.74 | .43 | 17 | 3.90 | .39 | -1.38 |
| Mothers’ coherence | 59 | 4.19 | 1.29 | 17 | 3.94 | .90 | .75 |
| Mothers’ positive comments | 59 | 3.92 | 3.11 | 17 | 5.47 | 3.41 | -1.78 |
| Fathers’ emotional availability | 50 | 4.75 | 1.07 | 16 | 5.45 | 1.00 | -2.28* |
| Fathers’ resolution with child diagnosis | 51 | 3.79 | .38 | 15 | 3.99 | 2.40 | -1.98 |
| Fathers’ coherence | 52 | 4.05 | 1.38 | 16 | 4.50 | 1.56 | -1.10 |
| Fathers’ positive comments | 52 | 2.54 | 2.56 | 16 | 3.00 | 2.28 | -.65 |

**Supplementary Table 3**

*Parents’ Representations and Emotional Availability by Children’s Adaptive Behavior* (*N* = 66-77)

|  | Low adaptive behavior  (VABS < 70) | | | High adaptive behavior  (VABS ≥ 70) | | |  |
| --- | --- | --- | --- | --- | --- | --- | --- |
| Variable | n | *M* | *SD* | n | *M* | *SD* | t-test |
| Mothers’ emotional availability | 65 | 4.99 | .95 | 11 | 5.95 | .57 | -3.25** |
| Mothers’ resolution with child diagnosis | 65 | 3.76 | .44 | 11 | 3.88 | .25 | -1.36 |
| Mothers’ coherence | 66 | 4.15 | 1.23 | 11 | 4.05 | 1.08 | .26 |
| Mothers’ positive comments | 66 | 4.11 | 3.28 | 11 | 5.27 | 2.61 | -1.12 |
| Fathers’ emotional availability | 66 | 4.78 | 1.08 | 8 | 5.95 | .45 | -5.50*** |
| Fathers’ resolution with child diagnosis | 58 | 3.80 | .36 | 8 | 4.07 | .29 | -2.05* |
| Fathers’ coherence | 60 | 4.18 | 1.43 | 8 | 4.00 | 1.46 | .32 |
| Fathers’ positive comments | 60 | 2.65 | 2.57 | 8 | 2.63 | 1.92 | .03 |

**p* < .05. ***p* < .01. ****p* < .001
